# Parasites or Cohabitants: Cruel Omnipresent Usurpers or Creative “Éminences Grises”?

**DOI:** 10.1155/2011/214174

**Published:** 2011-07-18

**Authors:** Marcos A. Vannier-Santos, Henrique L. Lenzi

**Affiliations:** ^1^Laboratório de Biomorfologia Parasitária, Centro de Pesquisas Gonçalo Moniz, Fundação Oswaldo Cruz (FIOCRUZ), Rua Waldemar Falcão 121, Brotas, 40295-001 Salvador, BA, Brazil; ^2^Instituto Nacional para Pesquisa Translacional em Saúde e Ambiente na Região Amazônica, Conselho Nacional de Desenvolvimento Científico e Tecnológico/MCT, Rio de Janeiro, RJ, Brazil; ^3^Laboratório de Patologia, Instituto Oswaldo Cruz, Fundação Oswaldo Cruz (FIOCRUZ), Rio de Janeiro, RJ, Brazil

## Abstract

This paper presents many types of interplays between parasites and the host, showing the history of parasites, the effects of parasites on the outcome of wars, invasions, migrations, and on the development of numerous regions of the globe, and the impact of parasitic diseases on the society and on the course of human evolution. It also emphasizes the pressing need to change the look at the parasitism phenomenon, proposing that the term “cohabitant” is more accurate than parasite, because every living being, from bacteria to mammals, is a consortium of living beings in the pangenome. Even the term parasitology should be replaced by cohabitology because there is no parasite alone and host alone: both together compose a new adaptive system: the parasitized-host or the cohabitant-cohabited being. It also suggests switching the old paradigm based on attrition and destruction, to a new one founded on adaptation and living together.

## 1. Introduction

*“It is derogatory that the Creator of countless systems of worlds should have created each of the myriads of creeping parasites and slimy worms which have swarmed each day of life*… *on this one globe.” *Charles Robert Darwin.

The words quoted above suggest that Darwin was rather concerned about parasites. If he had seen the hematophagous finches *Geospiza nebulosa*, also known as “vampire birds”, of Wolf Island in the Galapagos Archipelago [1], he would have presumably been overwhelmed. Many features of the parasitic life style can indubitably surprise most people regardless naturalist or not.

## 2. Parasitism and Symbiology

The term parasite (Latin *parasites *− Greek *παράσιτος*-*parasitos*, ***παρα*-** (*para-*, beside) + ***σίτος*** (*sitos*, wheat, food) “person who eats at the table of the bystander” “feeding beside”) is employed here in the traditional sense, but it must be stated that such concepts are only communication tools to be used in a flexible and relative way, as are the biological phenomena. In the words of van Beneden, the differences among parasites, mutualists, and free-living organisms are “almost insensible”, and according to Noël Bernard there is “no absolute distinction to be made” between *symbiosis and disease *[2].

From the study of lichens derived the concept of “consortium” to express the associations between phylogenetically distinct organisms that ranged from the loosest to the most intimate and essential, and the most antagonistic and one-sided to the most beneficial for the well-being of the both associates [3]. Albert Bernhard Frank (1877) at Leipzig coined the word *Symbiotismus*: “We must bring all the cases where two different species live on or in one another under a comprehensive concept which does not consider the role which the two individuals play but is based on the mere coexistence and for which the term *Symbiotismus* is to be recommended.” Though Frank's studies were well known, and he became one of the chief advocates of the view that many associations involving microorganisms could not be labeled parasitism, the term “*symbiosis*” was credited to Heinrich Anton de Bary to describe organisms coexisting or living together [3]. Thus we attempted to avoid the misleading aspects of *strictu sensu* definitions such as mutualist, pathogen, or parasite, used to characterize species which flow in a role-exchanging dynamic continuum [2–5], compared to a marriage, where it is difficult to measure the gains of respective partners [2].

 The term “*cohabitant*” is often more accurate than *parasite* [6], because it is increasingly clear that every living being, from bacteria to mammals, is a consortium of living beings [6, 7]. Even we propose that the term parasitology should be replaced by *Cohabitology because there is no parasite and host alone: both together compose a new adaptive system: the parasitized-host or the cohabitant-cohabited being* [6]. Symbiosis is a cyclical and permanent phenomenon in evolution [8]. The ubiquitous symbiotic consortia played a pivotal role in the prokaryote-to-eukaryote transition, and the term “*symbiogenesis*” was coined by Konstantine Merezhkovskii to designate “…the origin of organisms through combination and unification of two or more beings” [3]. Therefore, symbiosis, which is often under laid by hostility [9], is a powerful source of biodiversity inside the *pangenome* [10]. According to this concept, the Pangenome is the common (collective) genetic system of all living organisms, the organic molecules, and their complexes (DNA-and RNA-containing viruses, plasmids, transposons, insertion sequences) involved in the storage and transmission processes of genetic information. In fact, genes are remarkably outnumbered by retrotransposons and other types of mobile elements [10–12]. The navigation of mobile elements in the pangenome of the living beings is made by *infectrons*. Tosta coined this term to encompass the broad array of exogenous DNAs that invade a genome and interfere with its structure or organization, and, therefore with its function [13]. In fact, infectious agents are everywhere and they dwell the uppermost of our individuality: our genome. Indeed, it has been recognized that about 40% of mammalian genome is composed of retrotransposons derived from retroviruses [13–15]. *Symbiology* plays a central role in ecology and on the overall understanding of Biology. Nowadays it is widely accepted that most if not all metazoan organisms and many microorganisms harbor different microbes, mainly prokaryotes. These may be harmless commensals, mutually advantageous mutualists, or virulent pathogens, depending on the milieu [16, 17]. Microbes may account for up to half the weight of insects such as termites [2]. Some of these cohabitants present their own endosymbionts which produce enzymes that breakdown lignin and cellulose. Over 90% of the plants are associated with mycorrhizal fungi which help absorbing nutrients, but may act parasitically, depending on the environment conditions [18–20]. 

Most if not all known species are involved in parasitism, acting as parasites and/or hosts. It is estimated that *circa* 80% of the known species, which are found in many different taxa, are parasitic [20]. Parasitology (Cohabitology) plays a central role in biological sciences, not only because parasites constitute the great majority of the living beings [21], but also because they regulate countless populations in numerous ecosystems.

According to Dr. J. R. Lichtenfels, “If every part of the Earth were to disappear magically except for parasites, one would still see the outline of the planet”. He also states that we need to understand the parasites because without the knowledge about the “enemy” and its strategies, we will hardly be able to win the battle [22]. Nevertheless, this bellicose view which dates from the times of Pasteur may have hindered the understanding of the innovative potential of these creative organisms upon life as we know [2, 3, 5, 6]. Microbes were and are still largely seen as agents of disease and death rather than dynamic factors in transformation and evolution [2]. As will be seen below, this belligerent point of view, common in the literature, may be driven by parasites themselves. 

About 374 parasite species infect the *Homo sapiens sapiens* being more than 300 agents of zoonosis [23]. This is not surprising if we keep in mind that among our 25.000 genes only about 1% is exclusively human and more than 99% homology was observed between the human and chimpanzee genomes [24]. 

In the *latu sensu,* Parasitology would focus viruses, bacteria, fungi, animals, and plants with parasitic way of life. For methodological rather than conceptual reasons, viruses, bacteria, fungi, and sometimes even protozoa are focused by Microbiology and its branches Virology, Bacteriology and Mycology. Thus these sciences present considerable overlapping. The modern Parasitology (Cohabitology) constitutes much more than a branch of biological sciences, congregating, in elegant and complex fashion, diverse areas of the knowledge for example, Zoology, Ecology, Pathology, Molecular Biology, Biochemistry, Epidemiology, Immunology, Systems Biology, and others, thus constituting a rich multidisciplinary collection. The parasitism phenomenon comprises an intricate web of interactions in which the parasite not only is fed, sheltered, and transported through its host, but is also able to significantly modify its physiology, behavior, and even direct the routes of its evolution. 

Symbiotic consortia involving bacteria and blue-green algae had probably originated, respectively, mitochondria and chloroplasts of the eukaryotic organisms. This perception arose from the similarities between microbes and organelles pointed out by Joshua Lederberg and further examined by Wakeford [2] and Margulis [25]. The prokaryote-organelle continuum is the main stream of eukaryotic cell evolution and even the eukaryotic cell nucleus may have had a prokaryotic origin [26, 27] or even a viral origin [28, 29].

Besides different types of endosymbiont, protozoa may be associated with ectosymbiont spirochaetes that function as undolipodia or locomotion structures, which may have given rise to cilia and flagella of the higher organisms [30]. It is worth mentioning that mitochondria as well as pathogens like *Listeria*, *Shigella*, *Rickettsiae,* and vaccinia virus induce the polymerization of actin tails in the cytoplasm of the “host” cell [31]. This “evolutionary scar” may have been useful only for pathogen spread at first, but now it is also used for organelle translocation [31, 32] as in the spermiogenesis regulation in *Drosophila* [33]. It was hypothesized that the infection vestiges of the bacteria of the spotted fever group would play a pivotal role in the origin of sexual reproduction [34]. Sterrer [35] also proposed that infection may have originated sex. It should be kept in mind that sexual reproduction is involved in parasite resistance (*vide infra*), and the parasite-host interface is frequently depicted as a red queen race [36, 37] but we would rather use the Sisyphus punishment metaphor. Sisyphus, founder and king of Corinth (or Ephyra as it was called in those days), was condemned in Tartarus to an eternity of rolling a boulder uphill then watching it roll back down again (the Sisyphus metaphor was previously used to depict the parasite-host interplay [38].) In these consortia both species strive to overcome the opponent strategies, keeping a dynamic adaptive balance. In this kind of dance, the cohabitants beings suffer adaptive epigenetic changes, that is, they never retain the same initial state as is expressed by the red queen metaphor. 

Symbionts can cross continuum between commensals, mutualists, and pathogens in both directions. Also, parasitic and predatory life styles may be exchangeable and sometimes overlapping. Therefore, it is necessary to analyze the ecology of these symbiotic associations in a broader and dynamic form [2, 3]. 

Protozoa of the Phylum Apicomplexa such as *Toxoplasma gondii* and *Plasmodium* sp. present an organelle of vegetal origin, bounded by four membranes, called apicoplast, which presumably appeared from successive phagocytic processes involving an algal cell, before these protozoa adopted the parasitic way of life. Today these compartments constitute a chemotherapy target [39]. Similarly many invertebrate species including filarid nematode parasites such as *Wuchereria bancrofti* were infected by the bacterium *Wolbachia pipientis *and depend on the prokaryote for optimal reproduction [40], and this bacterium enhances the *Tribolium confusum* male beetle fertility [41].

Lichens are generally regarded as a classical example of mutualistic symbiosis, but the very description of the dualistic nature of lichens in 1869 (“*Die Flechten als Parasiten der Algen*”, *Verhandlungen der Schweizerischen Naturforschenden Gesellschaft*—“The Lichens as *Parasites* of Algae”, Proceedings of the Association of Swiss Natural Scientists) by the Swiss botanist Simon Schwendener (1829–1919) clearly declares the parasitic nature of the fungi and compares the fungus to a “spider” that slaughters its “victim” since the algae are penetrated by suctorial hyphae, termed haustoria. Actually the algal cells react to refrain the hyphal invasion, but are eventually killed during the fungi saprobic feeding. The axenic cultivation of each partner is not always simple, but the algae are more easily isolated and maintained (less dependent). These facts point to the parasitic nature of fungi. Phylogenetic studies using small subunit ribosomal DNA (SSU rDNA) indicate that lichen symbionts arose from parasitic fungi, and that there is no general evolutionary progression from parasitism to mutualism [42]. These authors propose that neither mutualism nor parasitism should be seen as endpoints in the evolution, and symbiosis and mutualism may give rise to parasitism, causing human diseases [5, 43]. Interestingly lichens are classified taxonomically, although made-up of two distinct species belonging to different kingdoms. Therefore, two species form a third one. That may be considered symbiogenesis. Similarly, the parasite-host biocartel is suggested to be the target of natural selection [20]. These consortia may arise from infection and/or predatism and the symbionts may become strictly interdependent. Animals that feed on algae, such as *Elysia viridis*, may preserve functioning chloroplasts and perform photosynthesis, and these organelles can even multiply in the marine ciliate *Mesodinium*. This kind of consortium is so common among marine zooplankton that it was asked why the cows are not green? [44]. Presumably because they are not translucent, but this problem was solved, at least in part, by the giant clam, the bivalve *Tridacna maxima*, by the development of hyaline organs, which scatter sunlight to the neighboring aggregated photosynthetic dinoflagellates or zooxantellae [45]. Most corals present zooxantellae, and these microorganisms play a pivotal role in the energy flow in the reefs, ecosystems of rich biodiversity. 

Photosynthetic symbionts also nourish animal species such as *Hydra viridis *and *Convoluta roscoffensis. *Although the latter can be 15 mm-long, this turbellarian flatworm is devoid of functional pharynx and mouth, so, unable to perform heterotrophic nutrition, relies solely on their endosymbionts for survival [45]. In addition symbiotic microbes take part in blood meal digestion in the lice *Pediculus* sp., the triatomine bugs that transmit *Trypanosoma cruzi* among vertebrate hosts [45].

## 3. Parasitism Ecology

Since parasitology approaches the interactions among species and their environment (which may be our very bodies), it is considered an area of ecology [46]. In fact parasitism, as well as other types of ecological relations, is considered symbiotic consortia. Currently, the evolutionary biologists are beginning to recognize parasitism as an important creative force of biodiversity [47, 48]. 

When our societies moved from the hunter-gatherer way of life to farming, our populations reached much higher numbers, supporting many epidemics and keeping the sedentary humans in close contact with flock animals as well as with its feces, urine, tissues, and so forth [49]. Zoonosis such as plague, measles, tuberculosis, smallpox, leptospirosis, flu, and pertussis was acquired from domesticated/domiciliary animals such as rats, cows, pigs, chickens, ducks, and dogs. Infections from wild animals such as AIDS, schistosomiasis, leishmaniasis, tularemia, and many hemorrhagic fevers comprise important sources of emergent and reemergent diseases. 

Parasites are continually exploring new available ecological niches in our organism and therefore originating emergent diseases. Protozoa such as *Giardia* are increasing its prevalence due to the vacant niches left by helminthes, as a result of the more efficient antihelminthic compounds used in both humans [50, 51] and domesticated animals [52]. New vacant niches are eventually tried by parasites as in the reports of enteric parasitism of human beings by *Ancylostoma caninum *adult worms [53], as well as the human infection by monogenetic trypanosomatids [54, 55], normally found infecting insects. 

Emergent diseases can both regulate the biodiversity of the wild life and threaten human beings [56] as well as other animals. Species invading new areas that leave parasites behind and encounter few new parasites can experience demographic overgrowth and become a pest [57]. In addition, invasive plant species that are more completely free from pathogens are more widely reported as harmful invaders of both agricultural and natural ecosystems. Therefore, invasive plants' impacts may be function of release from of natural enemies, including pathogens, causing their accumulation [58]. This indicates that parasite loss in animal and plant species invading new areas may confer significant competitive advantage, rendering them pests menacing the new ecosystem biodiversity. Zoonosis such as brucelosis, leptospirosis, salmonelosis, tuberculosis, and echinococcosis can cause considerable mortality and morbidity to human beings. Tuberculosis control in British flocks may have resulted in reduced risk of human infection [59] but the cowpox infection protected humans from smallpox, helping Sir Edward Jenner develop its vaccine (“*vacca*” = cow). It is worth noting that about two thirds of the emergent diseases had a zoonotic origin [58]. Parasitic diseases such as the different types of malaria had probably evolved from primate or avian infections [60, 61]. The recent outbreak of avian Influenza in Hong-Kong, with some serious and even fatal human cases, brings to mind the pandemic of Influenza that caused the death of more than 20 million people and had an avian origin, whereas the emergent H1N1 appears to be derived from swine infections [62].

The cuckoo birds (cuculids as *Cuculus canorus* L.) are neither endo-nor ecto-parasites and may be much larger than its hosts, but it has a typically parasitic behavior, in which the females lay eggs in the nests of other birds that will feed its offspring, often causing the death of the original younglings. Thus these species act as parasites and parasitoids or necrotrophs [63] at the same time. The parasitic infection, frequently deleterious at the individual level, can not only be advantageous for the populations, but even for the organisms individually [64]. These ecological relations can evolve into advantageous balances for both partners. Trophozoites of *Entamoeba gingivalis* and *Trichomonas tenax*, found in the human oral cavity, can help controlling the bacterial populations. These, in turn, produce proteins that function as “vaccines” against pathogenic bacteria. Some bacteria of our intestinal flora produce compounds useful for our metabolism, including glycosylhydrolases required for the optimum digestive system functioning. Enteric bacteria also induce and regulate the expression of many genes in the gut, such as fucosyl transferase enzyme characteristic of mouse intestinal villi [65], colipase, which is important in nutrient absorption, angiogenin-4, which helps to form blood vessels, and Small proline-rich protein 2A (Sprr2a), that fortify matrices that line the intestine [66–68]. The Gram-negative anaerobe *Bacteroides thetaiotaomicron* even promotes the development of the intestine's submucosal capillaries network [69–71]. Stappenbeck et al. [67] determined that the Paneth cells were required for the induction of the capillary network. These cells respond to *B. thetaiotaomicron* by transcribing the gene encoding angiogenin-4, a protein known to induce blood vessel formation [68–70]. Microbial community plays also a role in the development of Gut-associated Lymphoid Tissue (GALT) and, particularly, in the B immune system [71, 72]. There is growing recognition that microbial residents of the gastrointestinal tract might be important for both our understanding and treatment of obesity. However, many questions remain to be answered about the possible mechanisms [69, 70]. The intestinal flora can be affected by nematode infection as *Angiostrongylus costaricensis*, in mice [73].

 The human gut may present 500–1000 bacterial species and the number of microorganisms associated to our mucosae can be tenfold higher than the total number of human cells (around ten trillion, 10^13^, summing up nearly 1.5 kg microorganisms). The pattern of the gut microflora is peculiar for each individual [74]. Whenever imbalanced, the intestinal flora can be highly harmful and thousands of children die every year due to bacteria such as enteropathogenic or enterotoxigenic *Escherichia coli *[75–77] (it should be noted that multiple pathogens are often associates simultaneously [78].) However, the normal intestinal flora presenting bifidobacteria and lactobacilli has great metabolic importance, including the vitamin production. In general the excrements of an animal have more of certain vitamins than its food, a fact that explains why so many species carry out coprofagy. Vitamin K (menaquinones) is produced by intestinal bacteria such as *Bacteroides*, *Eubacterium*, *Propionibacterium*, *Fusobacterium*, *Bifidobacterium*, *Lactobacillus*, *Clostridium*, *Enterococcus*, *Streptococcus,* and others. The lack of these bacteria in neonates kept in incubators or subjected to antibiotic therapy may lead to the deficiency of menaquinone-dependent coagulation factors and hemorrhagic disorders. 

The normal microflora can also confer resistance to infections by microbes such as *Salmonella*, *Yersinia*, *Listeria*, *Vibrio* spp., *Clostridium difficile *[72, 73], and even cancer [76]. Because of that, it is important to manage the flora composition to furnish therapeutic strategies using probiotic, prebiotic, and symbiotic approaches. In fact, the bacteria of the digestive tract are fundamental to the proper development of the mammalian immune system [72]. By helping in the development of the host immune system, the symbiotic bacteria are in fact aiding in the construction of their own niche, protecting themselves from both foreign competitors and possible detrimental attacks from their host [68]. Angiogenin-4, like other mouse and human angiogenin, is a member of the RNAse superfamily. In humans, two members of the RNAse family, eosinophil-derived neurotoxin and eosinophil cationic protein, exhibit antibacterial and antiviral activities [79, 80]. Angiogenin-4 was found to have microbicidal activity against the pathogenic Gram-positive bacteria *Enterococcus faecalis* and *Listeria monocytogenes*, reducing the populations of each of these bacteria by more than 99% after just two hours of Angiogenin-4 exposure [71]. 

The lactic fermentation by *Lactobacillus acidophilus* keeps the acid pH of the vaginal mucosa, helping to protect the organism from pathogens such as *Trichomonas* and *Candida*. Thus the excessive hygienization, particularly employing bactericidal products, may not only predispose to other infections but also select drug-resistant phenotypes. 

Pathogenic bacteria, such as *Pasteurella multocida*, inoculated by the bite of the Komodo dragons (*Varanus komodoensis*) help its hosts, killing evading preys by sepsis and subsequent bacteremia [81]. Analogously, the polyDNA viruses, inoculated together with eggs of the brachonid parasitoid wasps (*Cotesia *sp.) on the caterpillar (Lepidoptera), aid immunosuppressing and blocking the endocrine homeostasis of the larval host, granting the success of the hymenopteran. These necrotrophic parasitoids consume the less essential parts of its hosts, sparing particularly the nervous and circulatory systems, in order to allow a prolonged survival, consequently optimizing the development of the pathogens. Larvae of parasitoid insects such as *Nemeritis canescens* present an even more hateful behavior, using long jaws to attack individuals of the same species in a severe intraspecific competition [82]. 

Plants such as the *Ipomopsis aggregata* and *Gentianella campestris* attacked by herbivores or parasites present increased development [82]. The infection of *Spermophilus richardsonii* squirrels by the *Trypanosoma otospermophili* is normally harmful to hosts, but under a vitamin B6 (piridoxine)-deficient diet, the parasitized animals have increased survival and growth. Parasitic/cohabitant nematodes and cestodes can accumulate highly toxic heavy metals such as lead and cadmium, favoring the survival of the hosts in polluted environments [83–85]. Similarly, the plasmids are normally deleterious to the bacteria, but in the presence of antibiotics they can confer resistance [86].

Moreover, pathogens such as *Bacillus thuringiensis* and the parasitoid or necrotrophic hymenoptera are used in biological control of plagues [87]. In addition, parasites may be used in monitoring environmental pollution [88].

It is interesting to notice that mutualism can generate a great dependence between symbionts and often most of the endosymbiont DNA is transferred to the host nucleus. Lateral or trans-species gene transfers between prokaryotes and eukaryotes take place from organelle to nucleus or between diverse microbes [89]. Because of multiple genome fusions, the evolutionary trees or dendrograms obtained via conventional phylogenetic algorithms may be converted to “rings of life” explaining the origins of chimaeric eukaryotes [90]. More than 8% of the human genome had a retroviral origin [91] and maybe about 40 genes were transferred from bacteria [92]. Some of these retrotransposons may be associated with mammalian malignancies and autoimmune disorders [93], but can be otherwise beneficial (*vide infra* placental formation). Up to 17% of the *Escherichia coli* genome may had been transferred [94]. The parasitic protozoa *Trichomonas vaginalis*, *Entamoeba histolytica,* and *Giardia lamblia* also display bacterial genes [95–97]. 

Interestingly, horizontal DNA transfer may take place between a eukaryotic protozoan and mammalian and avian hosts. In infected human macrophage lines, rabbits, birds, and patients, the *Trypanosoma cruzi* minicircle sequences can integrate into the genome of the infected hosts [98–101]. This is the first evidence in the literature of lateral and vertical DNA transfer from a protozoan to host. Naturally occurring human infections by *T. cruzi* were documented, where mitochondrial minicircles integrated mainly into retrotransposable LINE-1 of various chromosomes [101]. The fact that integration occurred almost always into LINE-1 reveals another original finding, reproducing a secondary parasitic transfer into previous mobile genetic element (primary transfer) similar to what happens with SINEs. These are short sequences (typically 100–200 bp) that appear to be parasites of LINE elements. Alu elements, a type of SINE, comprise fully 10.6% of the draft sequence of the human genome [11]. Then Tc kDNA and SINE are hitchhikers of LINE-1. Probably, the occurrence of oxidative stress during the infection can act as a genotoxic stress that threatens the integrity of the genome, creating DNA double-strand breaks (DSB) that activate mobile elements, such LINE-1 [102]. Moreover, LINE-1 presents some sequence homologies with *T.cruzi* kDNA, favoring the secondary parasite insertion or transference. There is evidence that L1 retrotransposition may be involved in the origin of illegitimate rearrangements and may contribute to DSB repair and genomic instability in mice [99]. Other contribution of these original works on *T. cruzi* kDNA transfer to human genome, based on the endosymbiotic theory of mitochondria, is the rarity of the phenomenon of gene transfer from bacteria to eukaryotes via endosymbionts: fully 223 human genes were identified in the sequence that had the strongest similarity to bacterial genes, suggesting that the genes were imported from bacteria into vertebrate lineage. Reanalysis of these data left only about 40 genes with bacterial closest relatives, and it seems likely that this number will decline still further [11]. It is noteworthy that the kinetoplast is a portion of the single trypanosomatid mitochondrion, an organelle with presumed endosymbiotic origin. Different mechanisms have been hypothesized for the transfer of foreign DNA to eukaryotes, including phagocytosis, infection, and symbiosis [13]. 

We may presume that after acquiring Chagas disease in the Beagle voyage to South America (e.g., [102–105], but see also [106]) (there is a great debate about the chronic disease Darwin presented and there are several authors supporting the Chagas disease hypothesis (considering indicative symptoms and his own report of being attacked by triatomine bugs), whereas other possible maladies include anxiety, panic disorder with agoraphobia, hypochondria, arsenic poisoning, chronic allergy, Crohn's disease, lactose intolerance. The dispute would presumably only be solved with the PCR examination of his remains from the Westminster Abbey, which was not allowed.), Charles Darwin did not expect to become a trans-kingdom chimera, resultant of an evolutionary mechanism that only now begins to be understood. 

Similarly, *Agrobacterium tumefaciens* DNA is transferred to vegetal host cells leading to the formation of tumor-like galls [107], where the pathogen proliferates and this process may pose valuable biotechnological applications. These harmonious relations could have resulted from either predatism or parasitism events. Cultures of *Amoeba proteus* had been accidentally contaminated in 1966 with a bacterium that infected the protozoan with high virulence. In some years, however, this deleterious effect had been reduced and currently this protozoan depends on hosting the procaryote. Therefore, a parasitic symbiosis can originate a mutualistic one [2]. The opposite is also true as mutualists may become adversaries. The red billed oxpecker *Buphagus erythrorhynchus* removes arthropod ectoparasites such as ticks from large African mammals and even use their 360° sight to warn the host of advancing predators. Because of ingesting blood-engorged ticks, they gained a “vampirish taste” and learned to take blood from opened wounds, delaying healing. Similarly, the New Zealand parrot *Nestor notabilis* feeds on sheep ectoparasites, but whenever there is a food supply shortage, they feed on the host subcutaneous tissues [108]. Life is often not as harmonious as it seems. Organelles [109, 110] and gene *loci* [111] may compete within a single “organism” cells. 

It is interesting to notice that in many cases of symbiosis between bacteria and protozoa, the prokaryotes are found within vacuoles that do not fuse with lysosomes. Similar nonfusogenic parasitophorous vacuoles are observed in infections by Mycobacteria and *Toxoplasma gondii*. The free-living amoebae *Acanthamoeba* spp. can harbor and even increase the virulence of prokaryotes such as *Legionella* spp. and Mycobacteria [112–114]. The protozoan host may protect *Legionella* from *Bdelovibrio* (a microbial parasitoid/predator of microbes) attack, but under stress conditions it may digest the hosted bacteria. Mycorrhizae may also act as either mutualistic or parasitic symbionts, depending on environmental conditions [2]. 

Protozoan parasites as *T. vaginalis* can be parasitized by the pathogen *Mycoplasma hominis* [115]. This phenomenon is called “hyperparasitism” [116]. Similarly *Ancylostoma* sp. may be infected by *Giardia lamblia* [117]. In the words of Swift [118]: *“So naturalists observe, A flea hath smaller fleas that on him prey; and these have smaller still to bite ‘em; And so proceed ad infinitum.”*

Thus, the ecosystems today, like many societies, maybe somewhat like “dog-eat-dog” and the early ones were presumably a “microbe-eat-microbe” world. 

The types of symbiosis are in constant transformation; virulence of the parasites is always varying according to the infection strategies and environment, including the host organism. Parasitism is not always harmful to the host and, depending on the environment conditions, it can be beneficial for both symbionts [119], giving rise to mutualism [120]. To be parasitized can confer the host a competitive advantage upon other more susceptible organisms. Just like the European settlers involuntary made use of its pathogens to decimate their opponents [49], *Paramecium tetraurelia* uses the taeniospiralis bacteria endosymbionts *Caedibacter* as an armament against susceptible strains. Similarly, *Parelaphostrongylus tenuis* worms were differentially advantageous in the population competition between cervids of the North America [119, 121]. The dynamics of competing species such as rabbits (*Oryctolagus cuniculus*) and hares (*Lepus europaeus*) is determined by pathogens such as myxoma virus and the helminth *Graphidium strigosum*. Likewise the competitive success between the coleopterans *Tribolium confusum* and *T. castaneum* is determined by presence of the sporozoan *Adelina tribolli* [122].

 The parasites frequently cause greater morbidity and/or lethality in the new or accidental hosts than in the usual one, in which they have evolved (i.e., coevolved). Thus, domestic cats (*Felis catus*) infected with *T. gondii* can seriously threaten species that have remained geographically isolated as, for instance, wild beasts of Australia. 

Protozoa [123], viruses [124], and bacteria [125–127] can be of therapeutical utility. In the past malaria was used as a sort of treatment, called “malariotherapy”, for the neurosyphilis, that presented high mortality [128, 129]. Although the infection by *T. gondii* is associated with the formation of tumors, mainly in immunosupressed patients [130], the chronic infection can be antitumoral [131]. This parasite can even reverse the multidrug resistance of human and murine tumoral cells [132]. The medicinal leech (*Hirudo medicinalis*) that had been very useful in the past, saving many lives, has returned to use nowadays and helps preventing postsurgical venous congestion. However, these annelids require the aid of the bacterium *Aeromonas* for digesting blood meals [44]. This prokaryote in turn can provoke infections and even septicemia in the individuals submitted to the bleeding by the hirudine, being, therefore mutualistic for the invertebrate host and accidental pathogen for the vertebrate [133]. Other hematophagous parasites such as *Ancylostoma caninum* are studied aiming medical applications on the lucrative market of anticoagulants and can even have inhibitory effect upon human melanoma metastasis *in vivo *[134, 135]. 

The disequilibrium in the pathogen-host interface, which results from a long coevolution process, can generate pathological alterations such as allergies, asthma [136, 137], and autoimmune manifestations including type I diabetes [138–140] and systemic lupus erythematosus [141]. Some studies [142] indicate that the elimination of the intestinal helminthes promotes autoimmune diseases, such as ulcerative colitis, Crohn's disease, and perhaps multiple sclerosis [143], that remain rare in underdeveloped areas where intestinal parasites are highly prevalent. The chronic helminth infections can revert autoimmune disorders preventing Th1-driven self-aggressions [144, 145] by induction of antiinflammatory cytokines as IL-10 and TGF-*β* [146, 147] and protect humans from cerebral malaria [148, 149]. Thus these parasites may become commensals and/or mutualistic. In this regard about 80% of human B lymphocytes are associated to the intestinal mucosa and each meter of intestine produces about 0.8 g of IgA daily, approximately as much as a mammary gland during lactation. *Necator americanus* parasitism may be also converting into a mutualistic consortium with humans [150]. 

It is provoking to keep in mind that ecological terminology could be applied to man as well. Most authors consider the cuckoos as parasites since they are fed by other species, but for feeding on milk, honey, eggs, and tissues of other “solicitous” species (e.g., bees, livestock and vegetables that are consumed without killing the individual plant) or slaving dogs, hawks and eagles for hunting, as well as pigs or dogs for finding truffles do not we parasitize them for their particular feeding abilities? Although it may seem heretical, are we not somewhat parasitic? Similarly, we do not think of phoresis when watching a Western movie cowboy riding a horse or of commensalism for having puppies or kittens at home.

## 4. History of Parasites

Symbiosis and Parasitism certainly preceded the rise of the first terrestrial organisms. The first evidence of bacterial parasitism is one billion years old [151]. 

The most primitive mycorrhizae have been found in fossil fungi dating from 460 million years ago, and 400 million years old lichen fossils were documented and it is worth mentioning that fungi important to humanity, such as *Penicillium* and *Aspergillus*, derived from lichen-forming ancestors [152]. 

The perception of parasitic disease and the attempts to control it may have outdated humankind. The medical use of medicinal plants by chimpanzees in the wild may have resulted in the very first origins of herbal medicine [153, *vide infra*]. 

The knowledge on Parasitology, particularly on larger parasites, is also antique. Egyptian papyri from the period 2,000–1,500 b. C., including the papyrus discovered in 1862 by Professor Georg Ebers in Tebas, dated of 1,500 b. C., describe parasitism by intestinal helminthes and *Schistosoma haematobium *[154]. Manuscripts found in India and China, dating about 2,500 and 3,000 b. C., respectively, describe observations of parasitic diseases and presumably comprise the earliest medical texts known. Hippocrates (460–375 b. C.) described aspects of the malaria and hydatidosis. Hippocrates and Aristotle (348–372 b. C.) named the intestinal *Teaniae* cestodes (taenia (Gr.) *ταιν*ί*α* = ribbon), but the *Taenia* species that infect humans were only described in detail in 1758 by Linnaeus. 

Biblical texts presumably describe the Guinea or Medina worms *Dracunculus medinensis* as “fiery serpents”. It states that the Lord said to Moses “Make a fiery serpent and set it on a pole, and it shall be that everyone who is bitten, when he looks at it, shall live” (Numbers XXI, 6–8). According to a number of authors [45, 155], the historical removal of this parasitic nematode, with the aid of a wooden stick, may have originated the pictorial representation of the medicine symbol, the Aesculapius staff, the caduceus. 

The history of the discoveries in Parasitology, particularly in the field of Protozoology, was usually related to the development of light and electron microscopy techniques. The first cells seen by microscopy were not the cellulosic cell walls ((Latin) “*cellula*” = small cell or chamber) observed in the cork by Robert Hook, but bacteria and trophozoites of *Giardia lamblia* that Antoni van Leeuwenhoek (1632–1723), a skillful and inquiring draping shop owner in Delft, Netherlands, collected from his own feces and those of his horse. Leeuwenhoek also observed *Opalina*, *Nyctotherus* and oocysts of rabbit coccid(s). At this time, microscopy was considered a hobby rather than a *bona fide* scientific activity and the embryos of Parasitology, Microscopy, and Cell Biology were twin born. It is important to point out that parasites comprise valuable experimental models in different fields of modern Biology. The use of microorganisms to approach fundamental aspects of Cell Biology has been termed “Cellular Microbiology” [156] and many discoveries were made via studies on parasitic protozoa [157–159]. Discoveries such as meiosis, continuity of chromosomes, cytochromes and electron transport system, among many others, were made on parasite-focusing studies [155]. Viral infections prompted the development of cloning and transfection techniques leading to the birth of Biotechnology and Molecular Biology.

## 5. Parasites of History

“*Ingenuity, knowledge, and organization alter but cannot cancel humanity's vulnerability to invasion by parasitic forms of life. Infectious disease which antedated the emergence of humankind will last as long as humanity itself, and will surely remain, as it has been hitherto, one of the fundamental parameters and determinants of human history.*” McNeill in *Plagues and Peoples*, 1976 [160].

Parasitic and infectious diseases have played a profound role in the outcome of wars, invasions, and migrations and in the development of numerous regions of the globe, thus determining the course of history [161, 162]. The most notorious conqueror of history, Alexander the Great died at the age of 32 following a two-week febrile illness. Speculated causes of his death have included poisoning, assassination, and a number of infectious diseases including typhoid fever, malaria [163], or West Nile Virus [164], among other (not so great) hypothesized etiologies. 

It is noteworthy that sometimes morbidity may be more decisive in war outcome than lethality. A sick or slowly dying soldier will have to be cared for by the others often expending scarce resources and sick men maybe more vulnerable to die from wounding. That is why biological weapons are frequently intended to debilitate the health (keeping the victim alive) rather than killing. Numerous pathogens have been tested and used as biological weapons and the consequences of their use (including bioterrorism) are notorious. Interestingly, as for parasitic diseases resistance (*vide infra*), the natural selection of immune variants in our progeny may comprise a pivotal defense against bioweapons [165]. 

 Parasitic and/or infectious diseases are responsible for more deaths than disasters, catastrophes, and wars altogether. The Sumatra 2004 Tsunami claimed the life of about 225000, people and the Japan 2011 Earthquake and Tsunami death toll exceeds 10000, whereas malaria causes nearly one million deaths annually (WHO, World malaria report 2010) (about 781,000 deaths worldwide were estimated in 20009, but a remarkable subnotification should be considered in many regions World Malaria Report, 2010—World Health Organization, available at http://www.who.int/malaria/world_malaria_report_2010/en/index.html.), amazingly not reaching the headlines, not to mention posttsunami malaria and dengue or other slaughtering infections such as tuberculosis. 

 The bubonic plague, also known as black death, transmitted by rodents through *Xenopsylla cheopis* fleas, claimed about 25 million lives in Europe, corresponding to approximately half the deaths in II World War. The death of 1/4 of the European population including 1/3 of the English, resulted in deep transformations to the society. The decline of the feudal system was promoted by the death of millions causing the shortage of available labor and land under cultivation began to fall. Therefore, local lords and aristocracy began to lose wealth and power. After that Europe was ready to enter the renaissance [166, 167]. 

Even the Church paid its tribute. At least 6 popes, cardinals, and other clergymen have died due to malaria, then also called Roman fever. The death of priests forced the Vatican to speed up the ordainment and even women, whose participation in the Church activities had often been limited, had been authorized to give the last rites to the uncountable dying. Because of the high plague mortality in Southern France, the Pope consecrated the Rhône River so that bodies thrown in its stream could have a “Christian burial” [166]. Mortality among clergy members comprised a great embarrassment (Christianity sense of guilt is more psychologically damaging than the Muslim “insh Allah” [Arabic] God willing [168].) since diseases were then frequently seen as God's wrath sent as punishment for sin and immoral behavior. Religious fanaticism grew and gave rise to sects of Flagellants. Flagellants wandered throughout Europe whipping themselves, recruiting followers, urging people to be penitent and spreading plague during their wanderings. According to several authors, the rise in religious extremism hastened the splintering of the Catholic Church, strengthening of the Reformist movement and the growth of Protestantism as an alternate belief system [167, 168] (according to McGrew (1960) [168], the appearance of the reform bill during the 1832 cholera pandemic was not accidental. The strike of cholera in Europe was associated to a stern abomination between the classes. In France, as well as other countries cholera was particularly common among the working classes, which believed that the bourgeoisie conspiring with the authorities was poisoning them for Malthusian reasons. This feeling spread through Europe as rapidly as the disease *per se*.). 

Malaria killed emperors and Pontiffs. The knowledge of its treatment had a strategic role for the Church. For a long time, the quinine obtained by *Cinchona officinalis* cork, was a secret kept by the Jesuits and in the XVIII century Protestants refused to recognize its antimalarial properties, resulting in needless suffering and deaths [169]. 

The 1918 Influenza pandemics killed 21 million people, being responsible for three times more deaths than the World War I and almost the same as World War II. Interestingly it is believed that about 30 million people succumbed to typhus during World War I. It is noteworthy that Influenza killed so many people in only one year [170]. 

Comparatively tuberculosis killed 2- or 10-fold more people than the World Wars II and I, respectively. As we can see, humankind has suffered much more from parasitic and infectious diseases than from political and social conflicts worldwide. In the words of Sir William Osler (1849–1919) “Humanity has but three great enemies: *fever*, *famine* and *war*; of these by far the greatest, by far the most terrible, is *fever*”. These enemies correspond to the knights of the apocalypse who lead to the fourth knight: “death”. Rather than independent, these flagella of mankind are intimately linked. War produces hunger and pestilence. These last ones, in turn dictate the routes of development and the outcome of wars [161]. 

Colonizers and priests, besides swords and crucifixes, brought pathogens, such as smallpox virus, which devastated indigenous nations in the Americas. A deliberate attempt to cause epidemics occurred when the British troops supplied Amerindians with blankets used by smallpox victims [169]. In past, war outcomes were not determined solely by the best tactics or weaponry, but often by the nastier pathogens [49]. During Mexico invasion in 1519, 2/3 of the Cortés Spaniards were killed by the belligerent Aztecs, but afterwards about 12 million Aztecs including the Emperor Cuitláhuac perished from smallpox. Similarly, before the conquest of Peru by Pizarro in 1531, much of the Inca population including the Emperor Huayna Capac and his successor were killed by smallpox. It is estimated that the New World Indian population declined as much as 95% in the years following Columbus's arrival [49]. 

The decay of the powerful Roman Empire may have been related to malaria (“*mala*” + “*aria”* = bad air; flowers at the lapel or by the windows and doors were introduced because of the belief that diseases were transmitted by the air). During the first century b. C. the agrarian districts at the periphery of Rome witnessed the appearance of a malaria epidemic, then called “Roman fever”, that lasted 500 years. Mortality was so high among children that many men were brought from German tribes to compose the fearsome Roman centuriae [166]. 

The occupation of great part of Africa and India by European colonizers was impaired by the severe flagellum of diseases such as malaria, cholera, and yellow fever. Some African leaders had considered malaria a protection against the European invader [45]. The Portuguese expression “de cabo a rabo” very common in Brazil, meant South to North Africa (from *Cape* Town, South Africa, to *Rabat*, Morrocco), which was not entirely dominated because of the “microbial soldiers”. Similarly, *Toxoplasma gondii* was suggested to be useful to protect our planet from an eventual extraterrestrial invasion and therefore it should be preserved as a possible interplanetary “weapon” [171]. 

Besides the conspicuous effect of the high mortality due to several infections, parasitic diseases such as ancylostomosis may cause insidious and cumulative morbidity producing a great impact on the host, at both individual and population levels. The Caucasoid southern USA population was considered indolent, irresponsible, and even assigned as “poor white trash” [45]. In fact, most of these people suffered from hookworm infection by *Necator americanus* (*necator* (Latin) = killer). In this regard the Brazilian countryside man, skillfully depicted in the Monteiro Lobato, famous Brazilian writer, character Jéca Tatú, was considered lazy and was in fact ancylostomotic [172]. Southern US populations were also afflicted by typhus and malaria. 

The outcome of the American civil war should not come as a surprise. The number of soldiers killed in combat or from wounds was about 110,000, whereas about 224,000 people died from diseases. It is estimated that diarrhea and typhoid killed 35,127 and 29,336 Union Soldiers, respectively. Another 14,379 died of malaria and 9,431 of dysentery. 7,058 troops succumbed to smallpox and 5,177 were defeated by measles (war Casualties—Spartacus Educational http://www.spartacus.schoolnet.co.uk/USACWcasualties.htm). 

Similarly, 20,356 French died from wounds in the Crimean war, whereas 49,815 died from diseases and 196,430 were sick. Typhus and dysentery also affected French soldiers during the invasion of Russia by Napoleon and after the battle of Ostrowo, 80,000 out of 450,000 men were sick. Perhaps more important than the participation of the so-called “Jack Frost” or “general winter” in Waterloo, had been the attack of the “general *Rickettsia prowazekii*”, which claimed the life of numerous men. From the 460,000 troops that marched from France in 1812, only 6,000 returned from the four-month stay in Moscow [162]. 

Indirectly parasites also determined the trends of war. Sex evolved because of parasitic infections (*vide infra*) and the struggle for women often caused conflicts among people. From the mythological war of Troy, a dispute for Helen, to the nowadays Yanomami in Venezuela, men fight to get women, as many other animals do [37]. 

In the past, infections traveled by train or ship together with people or as *Yersinia* in the fleas on furs or riding clandestine rodents. Currently, clandestine mosquitoes carrying clandestine parasites take few hours' airplane flights to reach distant continents. For this reason, in the past, the epidemics followed the maritime routes or railways, but nowadays cases of different infections in the neighborhoods of the airports in nonendemic areas are common. The infection can take place in the very airplanes, as in the cases of malaria among passengers traveling between Switzerland and Germany, in an aircraft coming of the South America. The air-conditioning system of the commercial aircrafts also propitiates efficient virus propagation. The recent, simultaneous incidence of severe acute respiratory syndrome (SARS) in China and Canada, clearly demonstrate that, in a globalizing world, we need a globalized epidemiology [173]. 

The parasitism of vegetal organisms also had a great impact in the history of the humanity. The Peruvian fungus *Phytophtora infestans* that infects potatoes provoked a disaster in European economy in the period of 1845-1846. The hunger was so devastating, that more than a million out of about eight million Irish starved to death. This fact caused the Irish Diaspora and thousands of people migrated for other countries such as the United States. Among the moving families, were the Fitzgerald and the Kennedy [161]. The political and historical implications of the mentioned parasitism are obvious. Another plant pathogen is responsible for the tea drinking tradition in England, where tea and coffee used to be consumed in approximately equal amounts up to the middle IX century. The parasitic fungus *Hemileia vastatrix* drastically reduced the coffee production in countries such as Ceylon and then Brazil became the main coffee-producing country in the world. This was interesting for the Brazilian economy, but many British had to change their beverage habits [45]. 

Infections in vegetables by the ergot fungus *Claviceps purpurea* could provoke the ergotism known as “Saint Antonio's fire” in the Middle Ages. It could have caused the strange behavior of young girls leading to a brutal witch-hunt in 1692 in the city of Salem, USA ([174, 175], but see also [176]). After the trial, 20 innocent, ergot-intoxicated people were executed for the crime of practicing witchcraft. Effects of mycotoxins are usually more pronounced in children and the Biblical story of death of first-born (that received double rations) in Egypt during captivity of Hebrews (the tenth plague abated on the Egyptians), also may be related to the presence of fungi on food, since stored grain would go moldy and presented deadly mycotoxins. Interestingly, other plagues were possibly related to an outbreak of a vector-born disease [177]. The 3rd (lice) and the 4th (flies or gnats) plagues may be involved in the transmission of microorganisms, and the 5th (livestock struck by pestilence or murrain) and 6th (boils and blains that break in sores on man and beast) ones may comprise the veterinary and human infections, respectively. The epidemiological hypothesis for the plagues is based on an ecological disequilibrium leading to algae proliferation (1st plague) with the consequent alteration in the populations and behavior of frogs (2nd plague) and insects (often preyed by frogs), some of which vectors of infectious diseases. Unfortunately these facts are generally either obscured or overviewed in history books. Otherwise the governments might be more concerned about infections.

## 6. Parasitism and Society

“*The role of the infinitely small is infinitely large*” Louis Pasteur.

The impact of parasitic/infectious diseases to our contemporaneous society can be demonstrated by the social and economic losses due to about two-three million deaths worldwide every year and nearly 3/4 of the human population is infected by some sort of pathogen. It is estimated that 500 million people are infected with *Plasmodium*, resulting in over 2,000 deaths every day (98 deaths/h) mostly among African children (WHO, 2010). We usually underestimate the effects of parasites on the course of human evolution. The increased resistance to malaria, largely due to the higher frequencies of hemoglobin disorders such as sickle cell anemia or glucose-6-phosphate dehydrogenase deficiency in African populations, where the malaria is endemic, clearly demonstrates that primarily deleterious mutations may be favored in response to the parasite stress. Thus in the parasite-host interface, both sides maybe submitted to intense selective pressures. 

Humans have developed “weapons” such as the behavioral strategies (i.e., not relying on mutations favored by natural selection) including the use of natural or synthetic antiparasitic substances. However, as parasites generally present shorter generation times, they can evolve and adapt more quickly, thus making use of a much diversified, renewable and efficient “arsenal” of virulence factors. Therefore, it seems that we will never get rid of pathogens and always be faced with this “arms race”. Interestingly, the use of natural products to fight parasitic diseases, that is, *therapy*, may have preceded human beings. Chimps use plants such as such as *Aspilia ossabicensis*, *Aneilema aequinoctiale,* and *Vernonia amygdalina* to fight intestinal parasites [178–180]. Even foraging mammals may use plants to fight parasites [181]. These animals may avoid diseases by keeping distant from feces, but parasitic nematodes take a ride in the spores of fungi which are propelled to several meters away by bursting sporangia. 

Readily treatable diseases such as roundworm and hookworm infections affect one billion and 900 million people, respectively. These parasitic nematodes may be responsible for, respectively, 10,000 and 60,000 annual deaths, mainly in the poor countries (*vide infra*). Parasitic infections can drastically reduce the physical and mental development of children, as well as the productivity of adults [182]. Low physical and cognitive capacities render parasitized people less proficient and thus restricted to less remunerated occupations. It reduces their access to good health and sanitary conditions, increasing risk of new infections, maintaining a cruel vicious cycle of social exclusion. Infectious diseases are responsible for about 80% of the deaths in underdeveloped countries, but present a minor importance for the public health of the present days First World nations [183]. The pharmaceutical industries, therefore, have had little concern for the development of new drugs for the treatment of these diseases. Although about 90% of human diseases are caused by infective agents, less than 5% of the research and development are dedicated to the resolution of these infections [184]. Less than 1% of the drugs incorporated in our therapeutic arsenal in the last decade are directed to tropical diseases [185]. Without the involvement of the public sector, generating social justice and the ultimate escape from misery it will not be possible to save our “human capital” [186, 187]. The little interest on the so-called diseases of the poverty or “neglected diseases” is astonishing, considering that one out of five people in the world lives in conditions of absolute poverty. There is evidence that poverty diseases and its research is neglected even by reputed medical journals [188].

Parasitology is of great relevance particularly, but not only, to the countries of the Third World, where more than a million people die annually victims of infections, being half of that due to infantile malaria. It is estimated that far more than 50% of the world mortality and morbidity is due to infectious and/or parasitic diseases accounting for 16 million annual deaths. For comparative purpose, cancer kills approximately six million people annually. In this regard, at least 15% of the human tumors have a viral etiology [189] and parasites including helminthes may also promote tumors [190]. Besides the spoliation frequently associated to parasitism, *Trichomonas vaginalis*, *Schistosoma haematobium,* and *S. mansoni* can induce cancer in infected tissues. *T. vaginalis* infection, the main nonviral sexually transmitted disease in humans, favors the transmission of HIV and human papilloma virus [191]. However, parasitic infections remain considered diseases of poverty and thus receive less attention from governments and industries than less prevalent, but profitable, disorders, with much smaller social impact. 

Although widely employed, the expression “tropical medicine” is not conceptually accurate, since it focuses illnesses not restricted to the tropics. In the past, the so-called tropical diseases as plague, typhoid fever, and malaria were found throughout the world. Malaria has been a serious endemic disease in countries such as United States, Canada, and England. Several Shakespeare plays mention malaria, then called “ague”, which comprised an infection with high lethality in England until the end of the XVIII century. The first quinine trials were carried out in Essex, about 50 km from the center of London [192]. The current prevalence of many infections reflects the efficiency of the hygiene conditions and control measures rather than climatic or geographic properties [193]. However, the existing climatic and sanitary conditions in the majority of the tropical countries favor the advance of disorders of infectious and parasitic etiologies. The global warming by the emission of gases, such as carbon dioxide, is promoting the greenhouse effect or global warming. This phenomenon can increase the distribution of mosquitoes such as *Anopheles* sp., *Culex* sp., and *Aedes* sp., enhancing the incidence of malaria, dengue, and yellow fever, among other infections [194]. 

The unplanned urban superpopulation associated to the frequently poor sanitary and housing conditions in underdeveloped countries (a large extent in tropical climate), favors the proliferation of pathogens and vectors, constituting an important factor in the high prevalences of parasitic diseases in these regions. Due to the environmental impact of society, typically rural diseases such as schistosomiasis, malaria, and visceral leishmaniasis are getting urbanized in different countries. 

About 45% of the world population is concentrated in the urban environment, whereas in Brazil this percentage may reach 80% [195]. Dense agglomerations strongly favor the transmission of infectious agents, especially considering that sanitary conditions of the majority of the cities in the Third World remain as in Biblical or medieval times and, therefore, besides the emerging diseases, we are still faced with old-fashioned infections as leprosy, tuberculosis, dengue, and others. 

The indiscriminate use of microbicidal and insecticidal compounds (in medicines and hygiene products) has prompted the appearance of resistant organisms, hindering therapeutical and prophylactic measures. Organ and tissue transplants or blood transfusions facilitate the transmission of parasites such as *Toxoplasma gondii*, *Plasmodium* sp. *Trypanosoma cruzi*, *Leishmania* sp. as well as numerous viral infections [196–198]. 

Besides the direct impact of the parasitic infections on host health, parasitism can subvert immune mechanisms critical to resistance to other infections. African regions with high incidences of intestinal nematodes, such as roundworm, whipworm, and hookworm, present higher prevalence and severity of tuberculosis cases and HIV infections [199]. This effect cannot be attributed to the nutritional status. It is noteworthy that AIDS pandemic promotes the spread of opportunistic parasites such as *Toxoplasma gondii*, *Pneumocystis carinii*, *Cryptosporidium parvum*, *Leishmania* sp., and many others, which are often called opportunistic agents. However, the classification of such microorganisms as “opportunistic” was often inadequate and misleading, as some so-called “opportunistic” microorganisms can also cause disease in normal hosts [17, 200].

## 7. Parasites: Singular and Prevailing Driving Forces in the Evolution of Man and Society

*“We cannot fathom the marvellous complexity of an organic being; but on the hypothesis here advanced this complexity is much increased. Each living creature must be looked at as a microcosm−a little universe, formed of a host of self-propagating organisms, inconceivably minute and as numerous as the stars in heaven.” *Charles Darwin.

Microorganisms played a major role in the evolution of higher organisms, including the wise, “modestly” and redundantly self-denominated *Homo sapiens sapiens*. Curiously the human body presents at least an order of magnitude more bacterial than human cells [201]. The “human wildlife” may be comprised by 10 to 100 million species living in and on us [11]. We resemble “microbial quarters” rather than perfectly designed masterpieces. 

It is presently clear that *Helicobacter pylori* can cause the gastritis, peptic ulcers, and even neoplasy. An increasing number of chronic nosologic entities believed to have genetic and/or environmental nature as acute rheumatic arthritis, arteriosclerosis, multiple sclerosis, schizophrenia and Alzheimer's [202] had been implicated. Even obesity may have an infective etiology (infectobesity). At least eight types of obesity-inducing virus (*in animal*: canine distemper virus, Rous-associated virus type 7, Borna disease virus, scrapie agent, SMAM-1 aviary adenovirus; *in Human*: adenoviruses 5, 36, 37) have been identified in animals, especially poultry and mice. Studies on humans are far less convincing; however, two adenoviruses, Ad-36 and SMAM-1, have shown adipogenic properties [203, 204]. In addition to the viruses,* Chlamydophila pneumonia *(*Chlamydia pneumonia*) and the gut microbiota can contribute to regulation of fat storages [70, 205]. Viral infections also shaped human evolution and history [206, 207]. Interestingly a protein of a viral origin may mediate sperm-egg fusion in mammals [208, 209], and endogenous retroviruses play a pivotal role in both placental morphogenesis and suppressing the maternal immune response against the embryo [210–212]. These retrotransposon-like sequences which may account for over 40% of the human genome and had been termed “infectrons” [7, 13] as mentioned above. The retroelements profoundly manipulated our genetic inheritance, thus were termed “human genome sculptors” [213]. 

Different symptoms of infection may be a sophisticated physiological manipulation to promote parasite dispersal. Coughing, sneezing, and diarrhea effectively mediate the shedding and environmental dispersal of respiratory or enteric pathogens, respectively. Biting may spread the rabies virus from an infected animal. Even the immune response may be exploited in the parasite life cycles. Hypersensitivity reactions lead to scratching and scarifications permitting pathogen invasion of subcutaneous tissues and spread. The discomfort produced in the immune reaction to *Dracunculus medinensis* leads the definitive host to immerse the affected limb in ponds, where the juvenile form of the parasite can encounter offspring-copepod host [155]. Fever will promote vector-borne diseases via enhanced hematophagy by mosquitoes by both rising of temperature (thermotropism) and sweating which is associated with the typical smell (chemotropism) produced by the activity of skin-dwelling microorganisms such as staphylococci and *Corynebacterium*. 

The paresis and allopecia produced on mammals with visceral leishmaniasis may be useful for “serving” the sand fly a parasite-supplemented blood meal. A hairless, nonmoving animal will be far more suitable for insect “safe landing” and feeding. The protozoan even damages the insect gut enhancing its appetite [214]. Similarly, the bird parasite *Trichomonas gallinae*, usually transmitted via drinking water, produces polydipsia (chronic excessive thirst) in the infected animal. 

*Schistosoma *sp. eggs are laid within the vascular compartment and require the host immune response to reach the environment via feces or urine. Strikingly, *S. mansoni* uses development signals from hepatic CD4^+^ lymphocytes [215]. 

Ectoparasitism and parasite cleaning produced numerous mutualistic consortia among many vertebrate and invertebrate species. The act of grooming ecto-parasites assumed a very important social function among primates. Inducing endorphin release, this behavior can reduce tensions and therefore lead to intraspecific cooperation, ultimately promoting social behavior [216]. 

 Parasites may influence the host behavior in order to facilitate their reproduction and dispersal. Acanthocephala can reverse the negative phototropism of Isopoda and Amphipoda crustaceans rendering them more easily predated by the birds, which will then harbor the adult worms [217]. Similarly, the lancet fluke *Dicrocoelium dendriticum* programs ants to offer themselves as food for grazing herbivores such as lambs and cows [216].

The behavior of vertebrates may also be affected. Trematodes may drastically alter the swimming pattern of killifishes, which may expose their shinny bellies on the water surface and therefore are easily engulfed by sea birds [216]. One of the most amazing “alien-driven” responses takes place in rodents. These little quick animals are always alert and pragmatically scared by the presence (and smell) of feline predators. Nevertheless, when they are infected by *Toxoplasma gondii *they not only cease to flee but also may challenge death by approaching a cat, the protozoan definitive host. *Toxoplasma* may similarly modify human behavior [218]. Infected people may become more agitated, less neophobic, aggressive and more prone to take part in traffic accidents [219]. *Toxoplasma*-infected women show different personality profiles tend to be less moralistic, disregarding rules, and taking more risks [219]. It was even anecdotally suggested that the typical attitude of the British and the French might be due, at least in part, to parasitism by *T. gondii *since in the former population the seroprevalence is under 20%, whereas in the latter it is over 80% of the population [220]. Xenophobia and racial discrimination may have risen as primitive strategies to avoid alien parasites within alien populations.

Pathogen resistance may have promoted kin altruism [221] and kin selection may have played a role in the origin of monogamy [222]. In social insects, especially ants, it is likely that variation in polygyny is primarily driven by factors other than those responsible for variation in polyandry, but both may be under selection by parasitism in more complex ways than generally appreciated until now, requiring further thorough investigation [223]. Among humans, the more polygamous a society, the greater its parasite burden, though meaning of this observation is not clear [37, 224, 225]. This may be due at least in part to the higher male susceptibility to infections [226, 227]. In polygamous animals, the variation in number of sex partners is greater among members of one sex than the other [228]. Therefore, if a sexually transmitted parasite can recognize the sex of its host, the reproductive rate of that parasite is expected to be greater in the sex that potentially has the larger number of partners; given the preponderance of polygyny among animals [228], sexually transmitted parasites should be more virulent in males than in females [229]. Sex differences in parasite infection rates, intensities, or population patterns are common in a wide range of taxa [229]. Ecological view usually postulated that sex differences in parasite infestation were due to differences in the life histories of males and females, with one sex perhaps eating more or different prey, and thus ingesting more infective stages, or perhaps inhabiting an area with greater tendency to harbor parasites, such as a stream margin [229]. Another tradition explained the sex differences to parasite infections based on the sexual dimorphism that are important to the host-parasite relationship, such as endocrine-immune interactions and mortality or senescence rates [229–234]. Some of the most clinically important parasitic diseases of humans, including ascariasis, leishmaniasis, malaria, schistosomiasis, trypanosomiasis, and a variety of helminthes show significant differences in male and female infection rates [229]. Hormones in general and sex steroids specifically may affect the genes, immune responses, and behaviors that influence susceptibility and resistance to infection [230]. In addition to sex steroids, several other steroid hormones, including glucocorticoids, pituitary-derived peptide hormones, such as follicle-stimulating hormone, luteinizing hormone, adrenocorticotropin hormone, and prolactin may influence sex differences in infection [230].

## 8. Conclusion and Perspectives

There is a pressing need of “change the look” at the cohabitation phenomenon between humans and parasites. A better cohabitation will depend on a better comprehension of the relational mechanisms between cohabitant(s) and the host(s). The following papers and books will contribute to change the look perspective at parasitic phenomenon: Ferreira [46], Trager [234], Brooks & McLennan [235], Sapp [3], Lenzi et al. [236], Paracer & Ahmadjian [237], Tosta [7, 13], Bush et al. [238], Margulis & Sagan [239], Bushman [11], Moore [217], Combe [5] and Combes [240], Lenzi & Vannier-Santos [6], Ulvestad [38], Rollinson & Hay [241], Ward [242], Jablonka & Lamb [243].

The new look guides, according to Carlos Eduardo Tosta (personal communication), to switch the current paradigm to a new one. 

The current paradigm presents the following concepts: (a) infectious agents are enemies that should be destroyed; (b) the function of the immune system is to destroy the infectious agents and to maintain the organism free of them; (c) genomes of the host and the infectious agents are closed structures without relationship among them; (d) the vaccines should maintain the organism free from infectious agents; (e) theoretical base: *attrition* and *destruction*. 

Otherwise, the new paradigm proposes: (a) “infectious agents” [*cohabitant/symbiont*] are coevolutionary partners; (b) the function of the immune system is to maintain the molecular individuality of the organism and to promote its adaptation to the infectious agents; (c) the host and infectious agents genomes are connected by mutual activation and infectrons exchange [they live in the pangenome], and they are organized in *coevolutionary networks*; (d) vaccines should contribute to the best adaptation of the “infectious agents” [cohabitant/symbiont] to the organism*: adaptive vaccines*; (e) theoretical base: *adaptation* and *living together* [=*cohabitation*].

In conclusion, the cohabitation of cohabitant (symbiont) and cohabited being raises the emergency of a new and *complex adaptive system: the parasitized-host* [244]. There is no parasite without host and *vice versa*. The future understanding of the parasitism phenomenon (cohabitation) through the Systems Biology perspective, will need the cooperation among parasitologists, infectologists, immunologists, pathologists, experimental and theoretical biologists, ecologists, mathematicians, physicists, experts in artificial intelligence and in computation science, and others [245, 246]. There is still a long way ahead before we can understand the language of the parasites: *The parascript*. The parascript was formulated by Harold W. Manter, and referred by Brooks and McLennan [235]:* “Parasites-furnish information about present-day habits and ecology of their individual hosts. These same parasites also hold promise of telling us something about host and geographical connections of long ago. They are simultaneously the product of an immediate environment and of a long ancestry reflecting associations of millions years. The messages they carry are thus always bilingual and usually garbled. Eventually there may be enough pieces to form a meaningful language which could be called parascript—the language of parasites, which tells of themselves and their hosts both of today and yesteryear.”*

*“We can be born 100% human, but we will die 90% bacterial—a true complex organism.” [247].*

*“The cohabitation symbiont-cohabited host is an unfinished symphony, so fascinating as Schubert's unfinished symphony. It never will have an end. This symphony is continuously played in two different tones: one is harmonious (Gaia tone) [248] and the other, noxious and dramatic (Medea tone) [242].”*
